# Diet composition and energy intake in humans

**DOI:** 10.1098/rstb.2022.0449

**Published:** 2023-10-23

**Authors:** R. James Stubbs, Graham Horgan, Eric Robinson, Mark Hopkins, Clarissa Dakin, Graham Finlayson

**Affiliations:** ^1^ School of Psychology, Faculty of Medicine and Health and; ^2^ School of Food Science and Nutrition, Faculty of Environment, University of Leeds, Leeds LS2 9JT, UK; ^3^ Biomathematics and Statistics Scotland, Rowett Institute, University of Aberdeen, Foresterhill, Aberdeen, AB25 2ZD Scotland, UK; ^4^ Institute of Population health, University of Liverpool, Liverpool L69 3GF, UK

**Keywords:** macronutrients, energy intake, energy density, energy balance regulation, obesity, hedonics

## Abstract

Absolute energy from fats and carbohydrates and the proportion of carbohydrates in the food supply have increased over 50 years. Dietary energy density (ED) is primarily decreased by the water and increased by the fat content of foods. Protein, carbohydrates and fat exert different effects on satiety or energy intake (EI) in the order protein > carbohydrates > fat. When the ED of different foods is equalized the differences between fat and carbohydrates are modest. Covertly increasing dietary ED with fat, carbohydrate or mixed macronutrients elevates EI, producing weight gain and vice versa. In more naturalistic situations where learning cues are intact, there appears to be greater compensation for the different ED of foods. There is considerable individual variability in response. Macronutrient-specific negative feedback models of EI regulation have limited capacity to explain how availability of cheap, highly palatable, readily assimilated, energy-dense foods lead to obesity in modern environments. Neuropsychological constructs including food reward (liking, wanting and learning), reactive and reflective decision making, in the context of asymmetric energy balance regulation, give more comprehensive explanations of how environmental superabundance of foods containing mixtures of readily assimilated fats and carbohydrates and caloric beverages elevate EI through combined hedonic, affective, cognitive and physiological mechanisms.

This article is part of a discussion meeting issue ‘Causes of obesity: theories, conjectures and evidence (Part II)’.

## 1. The role of energy intake in human energy balance regulation

Considerable interest has focused on energy intake (EI), expenditure and storage in attempts to understand obesity development and if or how this constitutes dysregulation of energy balance (EB). Enquiries have intensified, in parallel with the growing obesity epidemic, in search of theoretical explanations for obesity development that provide mechanistic solutions for prevention and treatment. With some significant exceptions (e.g. the fields of ecology, agriculture and physiological psychology), the study of ingestive behaviour in animals and humans and the study of human EI, metabolism and storage (body composition) have largely developed in distinct theoretical domains until the mid-late twentieth century [1].

By the late twentieth century, there was a growing view that that increased population-level obesity development is primarily owing to an excessive EI rather than decreased physical activity [2]. This led to renewed interest in the search for factors regulating the intake of energy and macronutrients (fat, protein, carbohydrate (CHO) and alcohol) and factors that perturb this regulation as mechanisms underlying the obesity epidemic [2–5]. Several theories had been developed to explain how almost every component of the EB system may function as a negative feedback homeostatic signal to influence food and EI. These include EB regulated around the needs for heat production, temperature regulation [6], expenditure or stores of energy (energostats) [7–9]. Kennedy famously proposed that body fat exerts negative feedback affecting both EI and energy expenditure (EE), through the process of lipostasis [10]. Several models have suggested that CHOs exert negative feedback on shorter-term EI. These include Mayer's glucostatic hypothesis [11], Russek's conceptualization of glucose status being monitored by hepatostats in the liver [12,13], or Flatt's model in which whole-body CHO stores (glycogenostats) exerted negative feedback on day-to-day food intake [14]. Similarly it has been proposed that plasma amino acid concentrations exert negative feedback on short-term EI (aminostasis) [15] or that this function occurs over longer periods owing to negative feedback from fat-free mass (protein-stats [16]), or a metabolic and motivational drive to eat to achieve a ‘target-protein intake', presumably related to requirements (protein leverage) [17]. It has also been proposed that body weight itself is more-or-less regulated (gravistat, [18], ponderostat, [19,20]), around set or settling points [21]. There have also been models to explain how specific nutrients may trigger overconsumption (energetic hyperphagia) and obesity development such as the current CHO-insulin model [22], itself preceded by Geiselman and Novin's sugar-induced [23] or Sclafani's CHO-induced hyperphagia models [24,25]. The sugar-induced hyperphagia and CHO-insulin models argued that CHO ingestion (particularly disaccharides, especially sucrose), produces hyperinsulinaemia, which then precipitates hypoglycaemia and a shift towards fat storage, elevating motivation to eat and body weight.

Dietary macronutrients summate to determine voluntary EI via sensory/hedonic cues, signalling along the gut-brain axis, [26–28], at the pre- and post-absorptive levels, or both [29,30]. The ingestion of macronutrients releases a battery of primary and secondary messengers that include hormones, amines, peptides and other neuromodulators that link the pre- and post-absorptive events related to macronutrient ingestion to the various interacting regions of the brain that are concerned with feeding and other motivated behaviours [31–33]. The central nervous system monitors metabolite levels, and endogenous substances produced by nutrient ingestion, directly from the blood via receptors and the array of hormones and other peptides released in association with nutrient absorption across the gut wall.

Neurobiological studies give a comprehensive account of how hedonic, reward-based pathways are far more central to EB homeostasis than was previously supposed. This helps to explain why eating behaviour can so easily lead to a chronic positive EB, given current environmental circumstances, which offer numerous facilitatory cues and few constraints regarding what, when and how much to eat. Macronutrients can act as very powerful unconditioned stimuli that modify the feeding response determined by previous conditioning to form learned food preferences that affect feeding behaviour. They can act as powerful sensory cues related to pleasure, and pleasure is important in directing goal-oriented motivated behaviours such as eating [31,34–37]. The effect of macronutrients may also be related to other cognitions and motivations such as health beliefs and other aspects of affect [31].

## 2. How does appetite (motivation to eat) affect energy intake and does this change in altered states of energy balance?

The current authors argue that the balance of evidence suggests that EB is regulated, but that regulation is neither precise nor symmetric, as proposed by many early negative feedback physiological models of homeostatic regulation [1,38]. This does not mean that there are not physiological changes that oppose weight gain or loss, such as the composition and energy cost of weight change itself, changes in the metabolic requirements associated with a change in metabolic body size, or changes in the cost of weight bearing activities, all of which both accompany, and tend to mitigate, weight change [38–40]. Compensatory responses in EI to subtle changes in EB (i.e. of relatively small cumulative magnitude over time) appear imprecise [41,42]. However, the aggregate evidence from observational, epidemiological and experimental studies suggests that components of the EB equation show compensatory responses that are much more marked in response to pronounced negative than positive EBs over time [1,38]. As weight is gained over time, there is limited evidence of physiological or behavioural systems actively working to prevent further weight gain. Changes in EE and its components play a significant role in response to energy deficits. Motivation to eat and EI appear to have a stronger influence on body weight regain compared with changes in EE, e.g. [43]. Responses to weight loss, both physiological and psychological, occur on a continuum influenced by factors such as the degree and duration of energy deficit, initial body composition and the psychosocial environment [38].

This suggests that appetite may be more physiologically controlled in negative EB situations, while weaker links exist between physiological functioning, food intake and motivation to eat in positive or neutral EB states [44]. The modern environment, abundant in energy-dense foods, is rich in sensory and environmental cues that influence eating behaviour and EI, presumably through hedonic and other affective mechanisms. Some authors argue that in resource-limited environments of our evolutionary past, hedonic and homeostatic systems were synchronized together to promote overconsumption during brief periods of food abundance, driven by environmental uncertainty. In an environment where food resources are unpredictable and finite, natural selection would have favoured overconsumption as an adaptive behaviour, limited (capability, opportunity) by environmental uncertainty [1,31,33,38,45]. There would have been less selection pressure for evolved systems that protect against weight gain as such an outcome would have been less likely in resource-limiting environments. This does not deny that the ‘energy homeostasis system' is relevant in modern environments. Modern environments have dramatically and rapidly changed relative to the environment in which EB regulatory systems evolved. It is also possible that EB regulation is not truly asymmetric, and that the current status of EB regulation on a population level may simply be a reflection of natural variation in an extreme (obesogenic) environment. If that is the case, we should conclude that EB regulation is incomplete, for the environment to exert such leverage in the first place. Nevertheless, like many complex traits, there is likely to be variation in ability to regulate or resist positive EB. Therefore, there is a need to understand the symmetry/asymmetry and strength of EB regulation, the time course of such regulation, and the impact altered states of EB have on homeostasic and hedonic cues for food intake in a rapidly changing environment. There is still much to be learned about phenotypes that apparently regulate EB precisely in the face of an environment that is conducive to excess EI, and to understand why some people appear to be resistant to weight gain in such environments. Speakmans *et al*.’s [42] considerations of set points, settling points and alternative theoretical models offer a useful framework in which to consider these issues.

It is often implied (but not explicitly stated) that eating behaviour is regulated as part of a feedback loop in EB regulation. However, in modern environments, there is little justification for eating behaviour related to weight gain to be regulated in the same way as described by traditional homeostatic models. Eating behaviour may exhibit stable patterns over time owing to habit, but these patterns may differ from the regulation described in homeostatic models of regulation [1,31,33,34,45].

Studies that attempt to elucidate mechanisms of appetite control primarily focus on short-term investigations of ingestive behaviour, emphasizing changes in motivation to eat or eating behaviour, rather than long-term studies. These short-term experiments assume that changes in motivation to eat or eating behaviour will translate into long-term EB regulation. However, the duration of these experiments (minutes, hours or days) is much narrower than the relevant time frame for such EB regulation to occur (weeks and months). In other words, researchers may be looking for evidence of EB regulation within a time frame where it is unlikely to happen [1]. Therefore, it is important to differentiate between longer-term mechanisms of EB regulation and shorter-term mechanisms that affect motivation to eat or EI. These considerations apply to the section below examining the idea that CHO stores or metabolism would regulate EB. The vast majority of studies examining the differential effects of macronutrients on EI are primarily acute/short-term studies. Few studies that have examined the impact of changes in dietary macronutrient composition on motivation to eat and EI over periods longer than a few weeks and most are limited to within or between-day studies. There are currently economic and methodological factors that limit such long-term studies [46,47]. However, given that EI and in turn appetite control are unlikely to be tightly coupled to EB regulation (be it symmetric or asymmetric) some caution should be exercised when taking inferences from acute or short-term studies. This also raises the issues of how tightly EB is regulated.

Unlike many physiological systems, which are regulated over periods of minutes, hours or days, body weight and composition are tolerant of considerable perturbations over weeks and months [1,44]. Evidence suggests that in subsistence economies, seasonal body weight fluctuations of approximately 10% were commonplace [1]. In many wild animals, seasonal fluctuations in body weight and composition are not unusual [38,48,49]. This does not mean that there is no regulation of EB, but that the nature and time-course of such regulation may be different to physiological regulation that is typically controlled by symmetrical negative feedback loops proposed by macronutrient-specific models of EI and EB regulation [1,38]. It is worth considering some examples of macronutrient-specific negative feedback models of appetite and EB regulation in the context of current debates about diet composition and obesity. The following section considers the historical context of CHO-specific models of appetite and EB regulation in detail because these models have dominated discussions of diet-induced obesity in the last few decades, but many of the regulatory principles under discussion, and their possible limitations, apply to other macronutrient-specific models concerned with protein or fat.

## 3. Examples of a carbohydrate-specific models of energy balance regulation via negative feedback onto energy intake

Mayer initially proposed that EB regulation is primarily controlled by short-term ‘glucostatic' responses. However, if short-term regulation is disrupted, longer-term ‘lipostatic' regulation can correct it [11]. In 1963, Russek suggested the existence of glucose receptors in the liver and put forward the hepatostatic theory of EB regulation [12]. Flatt expanded on these ideas in 1985 with the glycogenostatic model of appetite regulation, which proposes that glycogen stores provide negative feedback on EB [14]. These models make similar predictions regarding feeding behaviour: (i) CHO stores or metabolism have a negative feedback effect on EI, so manipulating CHO status, such as oxidation or CHO stores, will reciprocally influence EB; (ii) diets high in fat but low in CHO will promote excessive EI; and (iii) achieving excessive ad libitum EI on high CHO diets should be challenging without conscious effort owing to the strong negative feedback arising from CHO status. To support these mechanisms, studies should demonstrate a high likelihood that changes in CHO status will have predictable effects on feeding behaviour consistent with the models' predictions [5]. As a historical context, the next section considers studies that have tested the predictions of CHO-specific negative feedback models of EI control. Similar models exist for protein leverage [16,17] and for lipostatic regulation of EB [10].

## 4. Do carbohydrate stores/metabolism exert powerful negative feedback on energy intake?

### (a) Prediction 1: carbohydrate stores or metabolism exert negative feedback on energy intake

Two studies using indirect calorimetry examined this prediction in detail. The first study involved nine men and investigated the effect of altering CHO balance on day-to-day food intake. [50] The researchers depleted CHO stores over the first 24 h, creating a net CHO balance difference of 2.45 ± 0.67 MJ, while keeping EB controlled. Ad libitum food intake was then assessed over the following 24 h. To achieve CHO depletion, a high-fat (85% of energy), low CHO (3% of energy) diet was used. Surprisingly, this extreme dietary manipulation did not impact the subsequent day's ad libitum EI compared to the control group. In a subsequent follow-up study, six men were investigated, with each participant being studied three times in 5-day experiments [51]. They were fed to energy requirements for 2 days and received the same EI as either a high-CHO diet (79% of EI), medium-CHO diet (48% of EI) or low-CHO diet (9% of EI) over a 48 h period. The researchers examined the impact of these interventions on food and EI on the fifth day. Despite a significant difference in net CHO balance of 4.99 MJ between the high and low CHO diets, there was no significant effect on EI (and hence EB) during the fifth day. Consequently, although there were substantial changes in diet composition and perturbation of fat and CHO balance, this had little influence on subsequent EI.

### (b) Prediction 2: diets high in fat but low in carbohydrate will promote excess energy intake

Several studies have indicated that increasing the energy density (ED) of the diet by covertly incorporating fat has resulted in elevated EI, but not the amount of food ingested, e.g. [52–55]. Conversely, decreasing dietary ED by fat reduction has led to lower levels of EI and modest reductions in body weight [52,56]. Two studies have demonstrated that when individuals had unrestricted access to manipulated diets with consistent energy and protein density but varying fat to CHO ratios, they consumed similar amounts of food and energy [57,58]. These findings have raised the question of whether the effects of dietary fat on appetite and energy EB are solely owing to its higher ED, or if fat possesses distinct weight-promoting properties that are independent of its contribution to overall dietary ED. Furthermore, these studies challenge models suggesting that increases in CHO oxidation and/or storage *per se* serve as powerful, unconditioned negative feedback mechanisms on food intake or EI.

### (c) Prediction 3: excess ad libitum energy intake on high carbohydrate diets should be very difficult to achieve without a conscious effort

This hypothesis was tested in a study involving six normal-weight men who were each studied twice for a duration of two weeks. During this period, they were provided unrestricted access to one of two covertly manipulated diets [59]. The diets had proportions of fat, CHO and protein as a percentage of energy, with fixed ratios of 22 : 65 : 13. The low-ED diet had a calorie content of 348 kJ 100 g^−1^, while the higher-ED diet contained 617 kJ 100 g^−1^. The participants were allowed to adjust the quantity of food they consumed but not the composition. EIs for the two diets were 8.56 and 14.56 MJ d^−1^, respectively, leading to weight loss on the lower-ED diet and weight gain on the higher-ED diet. Although the perceived pleasantness of the diets did not influence intake, the participants reported feeling significantly hungrier on the low-ED diet compared to the higher-ED diet (*p* < 0.001). These findings indicate that excessive EIs can occur in subjects on high-CHO, higher-ED diets when they have no choice in diet selection.

These within-subjects repeated measures design studies, using covert dietary manipulations, do not address the issue of food or nutrient selection, as participants were only able to adjust the quantity, not the composition, of the foods they consumed. These studies potentially uncouple learned behavioural responses from the physiological signals resulting from substantial dietary manipulations [60].

## 5. Do short-chain carbohydrates lead to preferential fat storage and weight gain? The carbohydrate-insulin model

The CHO-insulin model proposes that increased fat deposition in the body, resulting from hormonal responses to high glycaemic index CHOs, preferentially promotes fat storage and weight gain [22]. A lively debate contrasting this model to the ‘EB model’ which views overconsumption as primarily driven by highly palatable energy-dense foods, has progressed over the last few years [22,61–63]. Both models attribute weight gain to changes in diet composition but propose very different mechanisms. High glycaemic index CHOs can be overconsumed as demonstrated in our earlier studies in humans [57,64,65] and in studies in rodents [24,25]. Consumption of sugar-sweetened beverages is associated with weight gain in children and adults [66,67]. It is perhaps less probable that a complex goal-oriented motivated behaviour such as feeding (and therefore EI) in a mammalian species that shows a remarkable lack of dietary specialization, would be geared to, driven (in any direction), or heavily levered by any one dietary macronutrient *per se*, as proposed by several macronutrient-specific models [10–12,14,15,17]. That is unless, feeding behaviour in modern humans were driven to the edge of physiological requirements for protein, CHO, fat or energy which, by-and-large, it is not. Nevertheless, some studies show subtle changes in food preference and selection in response to short-term prior energy, e.g. [68] or protein deficits, e.g. [69].

## 6. Strengths and limitations of macronutrient-specific models of human feeding behaviour

The great strengths of macronutrient-specific models of feeding are that they are rooted in our understanding of physiology and provide testable hypotheses. Carbohydrate-based models of EB regulation have an intuitive appeal. These models seem to align with the understanding of dietary fat as a risk factor for weight gain [70–74], because these predictions are testable, and because the predictions of these models offer a potential avenue for manipulating EB through changes in diet composition. Experimental evidence partially supports these models but does not give sufficient explanation of the way the diet and its nutrient composition affect motivation to eat, EI and EB. This is partly because the macronutrient content of foods are tangled up with each other and the ED of those foods. It is impossible to alter the level of one macronutrient in the diet without simultaneously altering the proportion of dietary energy from the other two macronutrients. Furthermore, because the metabolizable energy coefficient of macronutrients is different, they contribute differently to the ED of foods and diets [75]. This raises three questions: what determines the ED of foods and diets (and have these determinants changed in the last 20 years), how does dietary ED affect EI, and how do macronutrients, independent of ED, affect EI? These questions should be addressed, as far as possible, under controlled laboratory conditions and in real-life, although there are methodological constraints to studies in both environments.

## 7. What are the major determinants of dietary energy density?

Twenty-two years ago, we examined the relationship between macronutrient and water content and ED in 1032 ready to eat foods from the British food composition tables [76] (excluding supplements) [60]. It has been suggested that between then and now the composition of foods available in the diet has changed. We have therefore repeated these analyses in a UK company (Slimming World) database of 66070 branded foods commonly available to UK consumers. Food composition data are taken from food labels. Here we report the current analyses, using regression models similar to those of 22 years ago. In this analysis, drinks consumed were omitted from the analysis and will be examined in a future publication.

**Figure 1.**
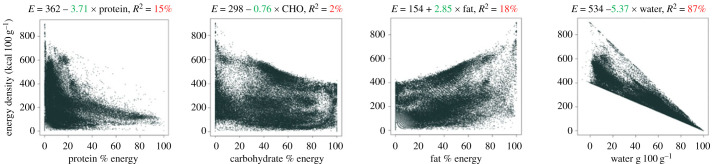
Relationship between percentage of energy from dietary macronutrients and water (in grams) (predictor variables) and energy density (kcal 100 g^−1^) of 66 070 ready to eat foods, taken from the Slimming World UK database of commercially available foods to consumers. Nutritional information is derived from food labels. All associations were significant at *p* < 0.001. (Online version in colour.)

When analysing the impact of water and nutrients (in g 100 g^−1^ of food) on dietary ED, it was observed that all nutrients contributed positively to ED, while water content had a negative contribution. However, the correlation between protein and CHOs and ED was generally weak, whereas fat and water showed stronger associations. When examining the relationship between nutrient composition (expressed as a percentage of total food energy) and ED, fat displayed a positive correlation with ED, whereas protein and CHO exhibited a weak negative correlation. The relationship for fat under these conditions is relatively weak (ED = 154 + 2.85 × fat, *R*^2^ = 18%). Conversely, the relationship between water and ED was much stronger and negative (ED = 534−5.37 × fat, *R*^2^ = 87%). Hence, although dietary fat contributes to higher ED, foods with high fat content (expressed as a percentage of energy from fat) do not necessarily have high ED (figure 1).

We have also examined the relationships between the percentage of energy from each macronutrient, the percentage of water and the ED of the diet reportedly consumed by 6155 individuals in 4-day food records from the National Diet and Nutrition Survey (2008–2014) [77,78]. The patterns in the data and the proportion of the variance explained are largely similar. The major determinants of the ED of foods and diets are the fat and more importantly, the water content. It appears that a ‘fat-water seesaw' determines dietary ED (when fat is expressed in grams, as per cent energy or in absolute kJ), which has implications for feeding behaviour. As the fat content of foods increases the water content tends to decrease. Some foods (e.g. commercially available snack products) are mixtures of fats and short-chain CHOs, with a very low water content. Fat has a lower osmotic load compared with CHO, which can potentially impact feeding behaviour [79]. This suggests that consuming a high-CHO or high-protein diet may lead to increased water intake, which could affect subsequent food consumption depending on how the water interacts with the food in the gastrointestinal tract.

## 8. How does altering the energy density of the diet by various means affect appetite and energy intake?

One series of studies has examined how, under the same experimental conditions, changes in the ED of the diet using primarily fat [54], primarily CHO [59] and primarily mixed diets [80] affects appetite, EI and EB in humans. These studies were conducted with participants having unrestricted access to covertly manipulated diets that remained consistent in composition throughout the experimental arms of the studies concerned. Subjects could only change the amount they consumed of systematically manipulated foods. Such covert manipulations limit individuals' capacity to learn about sensorially distinct foods, which may artificially inflate the casual effect of dietary ED on EI because it may preclude learned patterns of caloric compensation [60]. Taking quantitative-response studies at face-value may lead to an overestimation of the regulatory significance of passive over-eating when the ED of diets increases, while simultaneously underestimating the importance of active changes in food intake and subjective hunger. To explore potential differences in response to ED among three studies, a regression model was developed to identify indications of active decreases in food intake (excluding non-caloric drinks) and changes in hunger in relation to ED increases within the constraints of this experimental design [60]. The findings revealed that participants demonstrated more effective responses to changes in ED for mixed nutrient manipulations through active adjustments in food intake [80], followed by primarily CHO-based manipulations where detectable changes in hunger were observed [59], while primarily fat-based manipulations exhibited a lesser response. These findings align with limited evidence from infusion studies conducted on both rodents [81] and humans [82], which indicate greater caloric compensation in response to mixed infusions compared with glucose infusions, with lipid infusions resulting in the least caloric compensation. These results support the notion that different dietary macronutrients may have varying effects on satiety, with protein having the greatest effect and fat exerting the least impact [83–85].

## 9. How do dietary macronutrients affect appetite and energy intake?

There is evidence supporting the hierarchical effects of macronutrients on suppressing subsequent EI [55,83]. Protein has been found to be the most satiating macronutrient, followed by CHOs, and fat is the least satiating. This hierarchy applies to the macronutrients as they naturally occur in foods [60]. Under these conditions dietary fat contributes disproportionately to dietary ED. This hierarchy in the satiating efficiency of the macronutrients has been found in a number of contexts. De Castro's analyses of dietary intakes and feeding behaviour of free-living subjects, self-recording their dietary intakes, suggested that protein suppresses hunger and food intake in excess of its contribution to total EI. CHO suppressed hunger and EI roughly in proportion to its' contribution to EI. Fat produced less than caloric compensation [83]. Similar findings have been observed in short-term laboratory studies on appetite control, where protein has shown greater satiating effects compared with CHO [86–90], particularly when given at moderate and large amounts greater than approximately 1.5 MJ [91]. However, small manipulations below approximately 1.2 MJ often do not reveal differences in the satiating efficiency of macronutrients.

Several studies have found that high-fat, ED foods induce higher levels of EI than lower fat, less ED foods. This effect has been observed over periods ranging from two weeks [53–55] to several months [52]. It is important to note that in many of these studies, the diet is manipulated in a way that subjects can only change the quantity consumed while the composition of the foods remains fixed.

Limited research has directly compared all three macronutrients (at doses above 1.2 MJ) simultaneously. Westrate *et al.* suggested that when preloads exceed 1.2 MJ, macronutrients have varying effects on subjective satiety [85]. This hierarchy in the satiating efficiency of the macronutrients is evident in diet survey studies [60,83] and laboratory experiments [84], in relation to nutrient balance regulation [55] and post-absorptive metabolism in [82] humans and rodents [81]. The most tightly regulated macronutrients in terms of oxidation appear to be the most satiating.

For the purpose of this discussion, alcohol is excluded owing to its pharmacological effects, which can increase intake. Moreover, alcohol is primarily consumed in beverages rather than solid foods. In terms of energy content per gram, alcohol is second only to fat and therefore contributes to the higher ED of alcoholic beverages compared with their non-alcoholic counterparts. Protein and CHOs, on the other hand, tend to decrease the ED of ready-to-eat foods and are more satiating than fat and alcohol. Protein and CHOs are more effective at reducing the motivation to eat compared with alcohol and fat. Additionally, macronutrients and water can interact with each other, affecting ED and influencing the digestion and absorption of foods and nutrients. Is there any evidence to suggest that macronutrients independently affect appetite and EI, regardless of their contribution to dietary ED?

## 10. Do macronutrients affect appetite and energy intake independent of energy density?

Limited research has compared the satiating effects of macronutrients (protein, fat and CHOs) at equal ED in the diet. Van Stratum *et al.* [58] and Stubbs *et al.* [57] showed that high-fat and high-CHO diets with the same ED produced similar EIs over two weeks in 22 Trappist nuns or six Cambridge men, respectively. Some studies suggest that fat influences appetite and EI, albeit to a modest extent, regardless of its contribution to ED. CHO exerts a more immediate impact on satiety than fat [91]. A few studies have found this relatively subtle effect to be independent of ED, e.g. [92,93]. In line with this, a recent systematic review and meta-analysis of experimental studies found that studies which manipulated ED by varying the percentage of calories from fat versus keeping macronutrient composition constant to achieve diets differing in ED, produced similar effects on daily EI [94].

Fat has additional sensory impacts on food, providing moisture and mouthfeel, and acting as a carrier for fat-soluble volatile substances. These effects may also be unrelated to ED [95,96]. Evidence suggests that the sensory qualities of dietary fat and sugars can interact to influence the sensory pleasure response related to eating. Drewnowski initially demonstrated that combinations of sugar and fat appear to have a synergistic effect on the sensory pleasure response in human subjects, surpassing the effects of fats or sugars alone [95,97]. This finding has been supported by functional magnetic resonance imaging investigations, e.g. [98].

The differences between protein and other macronutrients have been consistently observed in studies comparing protein, CHOs and fat-enriched foods at the same ED. For instance, when individuals were provided with isoenergetically dense diets containing an excess of 4 MJ d^−1^ of protein, CHOs or fat, protein had a significant and substantial impact on reducing the motivation to eat compared with CHO and fat-enriched foods. The disparities between CHOs and fat could be attributed to CHOs exerting a more immediate satiety effect than fat [91].

In summary, when considering macronutrients in the diet (with fat contributing disproportionately to ED), there is a hierarchy in their satiating effectiveness. Even when controlling for ED, macronutrients have varying effects on appetite. Protein is significantly more satiating than CHOs or fat. Under controlled conditions, the differences between fats and CHOs are more modest. Furthermore, specific combinations of fats and CHOs may enhance the pleasure-related motivation to eat [98]. Limited evidence suggests that different types of CHOs and fats can have varying effects on satiety. However, these effects are modest and are most apparent in carefully designed and controlled experiments, potentially less notable under less controlled conditions.

Most work on the effects of different CHOs on EI has focused on unavailable complex CHOs or fibre. Various loads of fibre at one meal have been shown to decrease both hunger and EI at the next meal, but the effects are relatively modest [99].

While animal studies have demonstrated that altering the amino acid composition of proteins or protein-based preparations can have significant effects on feeding behaviour [29, pp. 277–331], there is currently limited systematic research conducted in humans, and this area remains overlooked. One reason for this is that systematically modified amino acid mixtures often have unusual or unpleasant tastes.

It is worth noting that attempts to systematically model the effects of physical, sensory and nutritional attributes of foods on motivation to eat and EI, across a large sample of common foods varying considerably in composition and in a large sample of subjects have been very limited. This is currently the focus of a ‘Satiety-Map' project at the University of Leeds.

## 11. What do we know about the relationship between energy density and energy intake from observational studies of free-living people, self-recording their dietary intakes?

Many dietary surveys using self-reported intakes, report energy and nutrient intakes but not food intake, making it difficult to examine relationships between ED (as EI/ weight of food eaten on a 24 h basis) and EI. It is important to examine both energy and food intake as outcomes since both factors give information about compensation of feeding behaviour. Twenty-two years ago, we examined the dietary determinants of EI in 73 weighed dietary records. Daily EIs were regressed against ED (ED), water (in grams) and each macronutrient expressed as a percentage of energy [60]. We have recently repeated these analyses in 6155, 4-day food records from the National Diet and Nutrition Survey (2008–2014) [77,78]. When the subject effect was ignored, the percentages of CHO and protein in the diet contributed negatively to EI but only explained small percentages of the variability in EI (3.2% and 3.3% for CHO and protein, respectively). The percentage of fat contributed positively to EI and the percentage of fat explained the largest proportion (9.7%) of the variability in EI of all the macronutrients. Water only explained 4% of the variability in EI in this study. The relationship between ED and EI, and ED and food intake were also examined. ED was associated with higher EIs explaining 6.9% of the variance and with lower food intakes, explaining 19% of the variance. Parenthetically, the relationship between ED and Association of Analytical Chemists (AOAC) fibre is small and the slope was negative explaining 5.5% of the variance, but that between EI and AOAC fibre in grams is positive, as for macronutrients and water. Compared to the macronutrients, fibre is a very small proportion of the diet in this analysis, rarely exceeding 35–40 g d^−1^.

**Figure 2.**
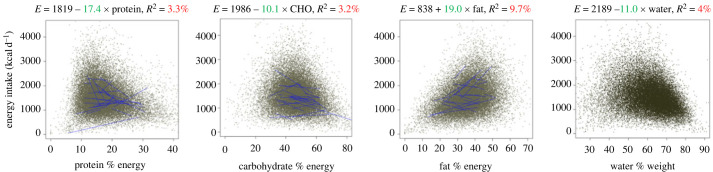
Relationship between percentage of EI from dietary macronutrients and water (expressed in g 100 g^−1^) and energy density of the diet (kcal g^−1^) (predictor variables) and total energy intake (kcal d^−1^). Analysis was derived from 6155 subjects self-recording their food intake by 24 h dietary recall over 4 days [77,78]. No corrections for plausibility of dietary energy intake were made. Sloping lines were a random sample of 40 individual regression lines, based on 4 days, to give an indication of inter-individual variation. All associations were significant at *p* < 0.001. (Online version in colour.)

**Figure 3.**
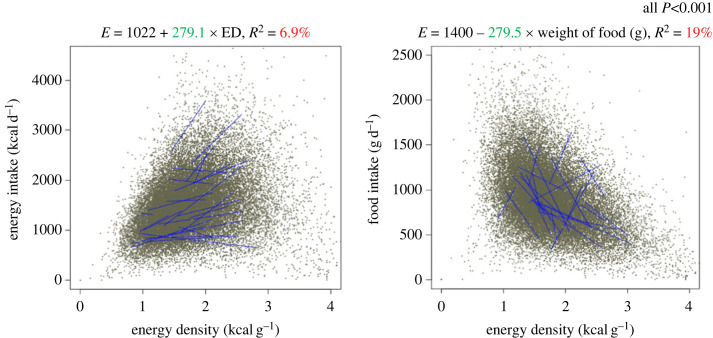
Relationship between dietary energy density (expressed in kcal g^−1^) (predictor variable) and energy intake (kcal d^−1^) and total food intake (g d^−1^). Analysis was derived from 6155 subjects self-recording their food intake by 24 h recall over 4 days [77,78]. No corrections for plausibility of dietary energy intake were made. Sloping lines were a random sample of 40 individual regression lines, based on 4 days, to give an indication of inter-individual variation. All association were significant at *p* < 0.001. (Online version in colour.)

There was considerable between-individual variability in the regression slopes for all of these relationships. When data were examined using mean-centred analyses (which has the effect of removing between-subject variability) the same patterns were evident, but generally the proportion of the variance in relationships explained was lower. It is not known if these patterns would be maintained in a longer set of observations (figures 2 and 3).

While the evidence from short-to-medium term laboratory studies suggests that increases in ED are more effective at increasing EI than at decreasing food intake; in longer term and the cross-sectional studies, we have conducted 22 years apart in different groups, in real-world environments, diets of higher ED appear more likely to be associated with smaller meal size or volumes (i.e. compensation in meal size for ED). However, this compensation appears incomplete because higher ED tends to be associated with higher EI overall. Longer-term experimental studies confirming these observations would be valuable [100]. Caution should also be exercised when interpreting self-report measures of dietary intake [46].

Returning to the discussion of models of EB regulation based on negative or indeed positive feedback from individual macronutrients, the evidence from a number of sources tends to suggest we should be developing more integrative multifactorial models to account for the effects of diet composition and ED on motivation to eat, EI and EB [60]. This raises the issue of ultra-processed foods.

## 12. Ultra-processed or highly processed foods

There has been a great deal of research and debate about the role of food processing in diet-induced over-eating [101–108]. Food processing is primarily the transformation of agricultural products into food, through mechanical, chemical or other technological approaches to change, preserve or improve the convenience with which a food is consumed. Processing ranges from the addition of preservatives, such as salt or sugar, forms of packaging and storage, including freezing and cooling of the use of protective storage environments, the addition of non-food additives and compounds to alter flavour, colour, texture or aroma [109]. Therefore, food processing causes a whole range of modifications that vary considerably, some of which may impact appetite and EI.

There are six named and one unnamed system which attempt to classify a degree of processing of foods, which differ in their categorization of foods [110]. The most commonly cited appears to be the NOVA system [111]. The NOVA system has four categories of degree of processing, ranging from minimally processed, through processed culinary components, processed and then ultra-processed foods. The NOVA definition of the latter is products obtained from formulations of several ingredients, like salt, sugar, oils, and fats, and substances, like flavours, colours, sweeteners, emulsifiers [111]. As pointed out by Visioli *et al*. [109], given the complexity of food processing, it is important to try and determine which of the many technological steps involved might be responsible for any potential negative or indeed positive consequences to health. Some highly processed foods are low in the density of micronutrients, fibre, water and dense in energy, fats, sugars and/or salt and additives, some of which influence the sensory attributes (taste, texture, aroma) of foods in a way which may stimulate EI. It is also the case that many such foods represent a large proportion of foods available on supermarket shelves.

It appears uncontroversial that many highly processed foods tend to be higher in ED than less highly processed foods [102,106]. It is critically important to understand from a mechanistic perspective, whether it is the ED, nutritional composition, non-nutritional modification and/or sensory attributes of foods, vis-à-vis, how they are processed that may or may not determine any associations between patterns of consumption and health outcomes [102,109,110]. Gibney and Forde highlight four observational studies as of 2022 that have examined the relationship between highly processed food, intake and health outcomes, using multivariate models that control for nutrient intake [110]. In three of the studies, when the model controlled for nutrient intake, correlations between highly processed food and disease outcomes remained, leading the authors to suggest that non-nutritional factors (food additives, packaging and of phytochemicals) may be involved in mechanisms of morbidity and mortality [110]. This is a complex and important area for future investigation, that was touched on in the symposium discussion, and relatively little is known about the impact on most non-nutritional food additives (or how they interact) to influence EI. Most evidence relating to food additives and health is toxicological for purposes of food safety [110]. Understanding how potentially complex interactions between many individually non-toxic compounds may or may not affect biological mechanisms of disease (in this case EI and obesity) is potentially challenging.

This article does not have sufficient space to address the several issues around both the definition of food processing or the relationship between highly processed foods and EI [102,109,110]. Briefly, it has been argued that highly processed foods may affect EI through their high ED, reward value and/or assimilability—i.e. the rate at which they can be ingested, digested and absorbed. However, as discussed in the next section, it may or may not be a useful communication heuristic to refer to foods as ‘junk', ‘ultra-processed' or ‘highly processed'. Some thought should be given to the socioeconomic and public health messaging implications of doing so. Consumer understanding of the effects of food production, provenance, processing and composition on health and well-being could be confused in an already crowded communication and marketing space. Collapsing multiple interacting mechanisms by which foods, their composition, physical structure and associated sensory attributes may affect EI, may not help us elucidate those biological mechanisms and may obscure, conflate or confound some of them. Systematic, structured dissection of the effect of nutritional and non-nutritional food attributes of foods on motivation to eat and EI is an important area for research development. The current authors argue that is important to systematically model the effects of physical, sensory and nutritional attributes of foods on motivation to eat and EI, giving careful consideration to the sample size and sample structure of both the population foods and population of humans being studied. We are currently attempting to develop these approaches in the Satiety Map project at the University of Leeds.

## 13. Dietary macronutrients, energy density and the neurobiology of human eating behaviour in the context of energy balance regulation

Appetite and EI are goal-oriented, motivated behaviours [31–33] operating in the context of EB regulation, which is both imprecise and over prolonged periods (i.e. not acute or short-duration studies), appears to be asymmetric [1,38]. The evolutionary forces that shaped those behaviours are unlikely to be those that directly shaped our current diet and its composition. Nevertheless, the integration of homeostatic and hedonic mechanisms humans evolved are likely to affect appetitive, eating and EB responses to the diet humans currently encounter. This potentially places our motivation to eat and food reward-driven behaviours in the context, and at times at odds with, other non-food related motivations and rewards (e.g. health, well-being). As Kringlebach, points out ‘food intake is driven by motivation and emotion which are in turn supported by reward and hedonic processing' and pleasure is a central cue to animals and humans that links food liking and wanting to patterns of learned ingestive behaviour [34, p. 309].

**Figure 4.**
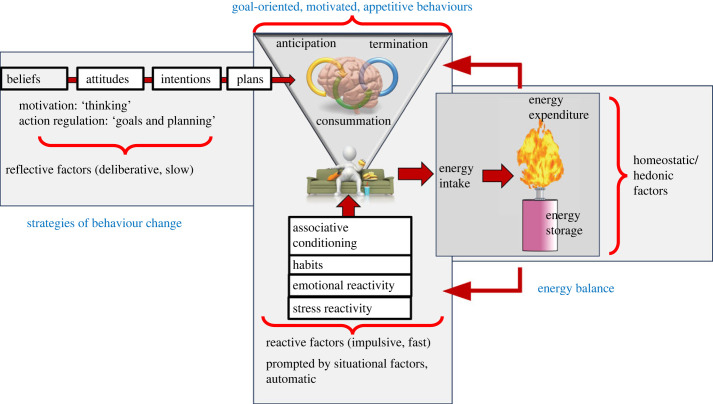
The central axis of eating behaviour, which like other goal-oriented motivated behaviours, operate in cycles of anticipation, consummation and termination. Directly linked to these cycles (in animals and humans) are reactive processes of learned habitual behaviours and response to environmental (e.g. food availability, palatability) and somatic cues such as stress and emotional reactivity. The neurobiological architecture of eating behaviour (i.e. selection and consumption of different foods) in turn, influences energy balance through the effects of eating behaviour on energy intake. The diagram acknowledges that components of the system exert feedback to influence both cycles of goal-oriented eating behaviour and the prompts and cues that influence reactive components of eating behaviour. Strategies of behaviour change, which involve cognitive modification of beliefs, attitudes, intentions and plans, aimed to reshape eating behaviour are often less influential than we would hope, particularly if they oppose reactive and/or homeostatic and hedonic factors. This schema assumes that the asymmetry of regulation evolved to align homeostasis and hedonics in resource-limiting environments, e.g. selection of energy-dense foods occurs because they are highly palatable, and pleasure is a central cue that links food reward to ecologically adaptive patterns of learned ingestive behaviour. (Online version in colour.)

Figure 4 depicts the central axis of eating behaviour, which like other goal-oriented motivated behaviours, operate in cycles of anticipation, consummation and termination. Directly linked to these cycles (in animals and humans) are reactive processes of learned habitual behaviours and responses to environmental (e.g. food availability, palatability) and somatic cues such as stress and emotional reactivity. This central axis shapes the architecture of eating behaviour (i.e. selection and consumption of different foods), which in turn influences EB through the effects of eating behaviour on EI. The diagram acknowledges that components of the system exert feedback to influence both cycles of goal-oriented eating behaviour and the prompts and cues that influence reactive components of eating behaviour, which are not necessarily within the realm of conscious awareness or control. Finally, and perhaps, often with less influence than we would hope, are strategies of behaviour change, which involve cognitive modification of beliefs, attitudes, intentions and plans, aimed to reshape eating behaviour. Those strategies may be less effective if they oppose reactive and/or homeostatic and hedonic factors. This schema assumes that homeostasis and hedonics are often aligned, owing to the asymmetry of EB regulation. For example, over-eating energy-dense foods which are highly palatable (often because they are energy-dense) is an example of goal-oriented, motivated appetitive behaviours functioning as they would be designed by natural selection in a resource limiting environment, even if the environment we currently inhabit is not resource-limiting. For many animals (including humans) pleasure is a central cue that links food reward to patterns of learned ingestive behaviour. Thus, the casual links between changes in components of the system variously highlighted by different theoretical models of the physiology of EI or EB regulation are likely to be more tenuous and complex than is sometimes assumed. Perhaps one of the key limitations of macronutrient-specific models that propose peripheral physiology exerts powerful unconditioned negative feedback on eating behaviour and hence EI, is that they underestimate the complexity of behaviour.

Because a great deal of human behaviour is both reactive and learned, it is possible that the environment can produce prompts, cues and stimuli that influence learned patterns of motivation to eat, e.g. [112]. It is frequently stated that the ready availability of cheap, highly palatable, readily assimilated, energy-dense foods is a major factor responsible for obesity development in modern environments. In fact, these are several factors which characterize the nutritional environment of modern humans and several potential mechanisms which may affect EI. The ED and macronutrient composition of foods has received considerable attention because many theoretical models of EB regulation are based on feedback from the macronutrient composition of foods, energy stores or expenditure. Availability, palatability, ED and assimilability (fast versus slow foods) of foods should have little impact on macronutrient-based negative feedback models if those models exert a powerful effect on EI. However, in a neurobiological model of eating behaviour operating in the context of imprecise, asymmetric EB regulation, each of these factors can have a large effect on motivation to eat, eating behaviour and EB [1,31,33,34]. Such models appear to help explain the relationship between appetite control, EI and asymmetric EB regulation, leading to the development of obesity in modern food environments, and the difficulties people have in losing weight and maintaining weight loss. By expanding our models to take account of the complexity of goal-oriented motivated behaviours, we may be able to dissect the several mechanisms that operate concurrently to influence diet-induced obesity in humans.

## 14. Conclusion and future directions

Several theories have been developed to explain how almost every component of the EB system may function as a negative feedback homeostatic signal to influence food and EI. The great strength of such negative feedback models is that they are rooted in our understanding of physiology and provide testable hypotheses. Many are macronutrient-specific. Experimental and epidemiological evidence partially supports these models but does not give sufficient explanation of the way that the composition of the diet affects motivation to eat, EI and EB.

As macronutrients occur in the diet, protein, CHOs and fat exert different effects on satiety or in the order protein > CHOs > fat. These relationships are partly intertwined with the contribution of each macronutrient to dietary ED. Dietary ED appears primarily to be determined by a fat : water see-saw in the composition of foods and diets. Increasing and decreasing the ED of foods and diets by various means tends to lead to corresponding increase or decrease in EI, which appear to be more (albeit incompletely) compensated for when learning cues are intact compared to covert manipulation studies.

There is some evidence that dietary macronutrients have different effects on satiety and EI independent of ED. Of particular relevance is the satiating effect of protein and the impact of short-chain CHOs, caloric beverages and mixtures of fats and sweet short-chain CHOs in elevating EI.

Appetite and EI are goal-oriented motivated behaviours operating in the context of EB regulation, which appears to be both imprecise and over long periods, asymmetric. Human EB regulation appears more tolerant of positive than negative EBs. Despite their theoretical elegance and parsimony, macronutrient-specific negative feedback models of EB regulation have limited capacity to explain how availability of cheap, highly palatable, readily assimilated, ED foods lead to obesity in modern environments. Neuropsychological constructs including food reward (liking, wanting and learning), reactive and reflective decision making, in the context of asymmetric EB regulation, give more comprehensive explanations of how foods containing mixtures of readily assimilated fats and CHOs and caloric beverages elevate EI through combined hedonic, affective, cognitive and physiological mechanisms. Understanding these multiple mechanisms, how they inter-relate and their importance in determining behavioural pathways leading to overconsumption and for managing therapeutic induced energy deficits, may expand our knowledge of obesity development and its behavioural management through more effective prevention and treatment options in the future.

In considering the effect of diet composition on appetite and EI in humans, we have discussed the limited evidence from primarily acute or short-term and largely cross-sectional observational studies in the real world, for which there is a paucity of data. The vast majority of studies on human appetite control are conducted over hours or days and may say little about long-term EB regulation [1]. These studies are limited in number, duration and extent of dietary manipulation. There are few prolonged studies that directly compare the effects of systematically manipulated diets on appetite and EI. There are even fewer studies of sufficient magnitude or duration to make statements about significant changes in EB. In the modern world a large proportion of over and undernutrition occurs in the ±10% range of EB. It would be valuable to conduct structured comparisons of interventions that achieve positive and negative energy imbalances to this extent in people with lower and higher body mass index.

There are considerable challenges in generalizing typical controlled feeding studies examining the effects of macronutrients on appetite, EI and EB, to real-world settings. Firstly, people do not usually consume systematically manipulated diets. They tend to select and ingest foods from literally tens of thousands available in the food supply. Patterns of food intake tend to be habitual [112,113], but there is far more noise in the nutritional environment of the real world than that of diet manipulation studies. Secondly, as mentioned in this paper, patterns of food selection and intake have a large, learned component which is likely to be central to acquired and often automatic behaviours that determine appetite control and patterns of EI [47,113–115]. Eating behaviour is shaped heavily by learned and anticipatory processes, such as associative conditioning [116]. This implies that understanding how food composition affects appetite and EI requires studies where patterns of learned associations can be established between sensory and nutritional, contextual cue-based characteristics of foods on the one hand and the physiological and hedonic consequences of ingesting those foods on the other. Food preferences and dietary habits are probably shaped by a number of additional factors not directly related to the physiology of EB regulation—e.g. culture, economics, pleasure, convenience. While, it is often claimed that laboratory measures and interventions concerning diet composition and EI afford greater control over both the intervention, the experimental environment and the outcomes being measured, that control may create biases and artefacts [47]. It is therefore necessary to pay some attention to observational studies where major gaps in the evidence remain.

There are also methodological problems and uncertainties associated with observational and epidemiological assessments of the relationship between food/diet composition and EI. A key problem being the measurement of food intake and EI itself, in which errors are high, non-random and assessments are biased by mis-reporting of dietary intakes [46,117]. In many studies, the population is not always representative of the general population. Correlational studies assume that relationships established in a particular study context are uniform over time, but they may not be. In both ‘real-world' and controlled studies, energy and nutrient intakes are often treated as primary outcomes but in reality they are secondary outcomes of observed/measured eating behaviour, which can be altered by other (sometimes unmeasured) influences such as food preference, avoidance, prior learned experience [47]. These concerns become greater the shorter the time-window of observation [47]. There is therefore a need for novel study designs that combine prospective cause-effect interventions, with parallel examinations of the same phenomena under less tightly controlled naturalistic conditions. The methodological constraints to such designs are considerable but not necessarily unsurmountable. New technologies, approaches to data analytics and wearable sensors may improve our understanding of food intake [118–121], patterns of physical activity and EE [122–126] and changes in body composition (and hence EB) in the environmental contexts were they occur, e.g. [127–129] to provide comparative measures across experimental environments (laboratory and real world) and to aggregate data across time windows relevant to behavioural trajectories that affect longer-term EB. These are exciting areas for future development that may help us better understanding patterns of behaviour (eating) that have a central impact on EB and health outcomes.

Aligning macronutrient-based feeding models with what we are rapidly learning about the neurobiology of eating behaviour, the intertwined nature of (asymmetric) EB homeostasis and hedonics, the reflective and reactive nature of human behaviour and the multiple, mutually reinforcing mechanisms that recruit goal-oriented motivated behaviours to select and ingest foods may lead to a greater understanding of diet-induced obesity. A more standardized and integrated approach to the future study of appetite and EI in the context of tracked changes in EB would be extremely helpful. There is a need to clarify and standardize the definition and construct-operationalization of both traits, e.g. [130,131] and states related to individual differences in eating behaviour and motivation to eat respectively. In particular, clarifying definition and measurement of human motivation to eat constructs such as hunger, appetite and satiety into forms of motivation [31,33,34,37,45,132]. Testing those measures against observable patterns of goal-oriented ingestive behaviours (anticipation, consummation, termination [37]) would be potentially informative. Modelling the nutritional, non-nutritional and contextual attributes of foods that are associated with motivation to eat or not eat would expand our models of food composition and EI into a more integrated, multifactorial models. Such models could then be tested in laboratory studies and real-world settings. Such approaches may help integrate theoretical mechanisms, align experimental designs, and develop and harmonize key measurements of EI in the context of tracked EB behaviours. Integrating motivational measures relating to eating with tracked EB behaviours and EB status, occurring in objectively recorded environmental contexts, over weeks and months, may improve our understanding of the relationship between the composition of the diet, motivation to eat and eating behaviour in the development, prevention and/or management of obesity.

## Data Availability

Data from the analyses presented in figure 1 can be obtained on request from Slimming World UK and provided by R James Stubbs. Data from figures 2 and 3 can be obtained from the following sources: [77,78].

## References

[1] Stubbs RJ, Tolkamp BJ. 2006 Control of energy balance in relation to energy intake and energy expenditure in animals and man: an ecological perspective. Br. J. Nutr. **95**, 657-676. (10.1079/bjn20041361) 16571145

[2] Prentice A, Black A, Murgatroyd P, Goldberg G, Coward W. 1989 Metabolism or appetite: questions of energy balance with particular reference to obesity. J. Hum. Nutr. Diet **2**, 95-104. (10.1111/j.1365-277X.1989.tb00014.x)

[3] Blundell J, Stubbs R. 1999 High and low carbohydrate and fat intakes: limits imposed by appetite and palatability and their implications for energy balance. Eur. J. Clin. Nutr. **53**, s148-s165. (10.1038/sj.ejcn.1600756) 10365993

[4] Blundell JE, Tremblay A. 1995 Appetite control and energy (fuel) balance. Nutr. Res. Rev. **8**, 225-242. (10.1079/NRR19950014) 19094287

[5] Stubbs RJ. 1998 Appetite, feeding behaviour and energy balance in human subjects. Proc. Nutr. Soc. **57**, 341-356. (10.1079/PNS19980052) 9793991

[6] Brobeck JR. 1946 Mechanism of the development of obesity in animals with hypothalamic lesions. Physiol. Rev. **26**, 541-559. (10.1152/physrev.1946.26.4.541) 21002972

[7] Booth D. 1972 Postabsorptively induced suppression of appetite and the energostatic control of feeding. Physiol. Behav. **9**, 199-202. (10.1016/0031-9384(72)90235-1) 4654732

[8] Friedman MI, Stricker EM. 1976 The physiological psychology of hunger: a physiological perspective. Psychol. Rev. **83**, 409. (10.1037/0033-295X.83.6.409) 1005583

[9] Langhans W, Scharrer E. 1992 Metabolic control of eating, energy expenditure and the bioenergetics of obesity, vol. 70 (eds AP Simopoulos, B Koletzko), pp. 1-67. Basel, Swiutzerland: S Karger. 1292240

[10] Kennedy GC. 1953 The role of depot fat in the hypothalamic control of food intake in the rat. Proc. R. Soc. Lond. B **140**, 578-592. 13027283 10.1098/rspb.1953.0009

[11] Mayer J. 1953 Glucostatic mechanism of regulation of food intake. New Engl. J. Med. **249**, 13-16. (10.1056/NEJM195307022490104) 13063674

[12] Russek M. 1963 An hypothesis on the participation of hepatic glucoreceptors in the control of food intake. Nature (Lond.) **197**, 78-80. (10.1038/197079b0) 13975629

[13] Russek M. 1981 Current status of the hepatostatic theory of food intake control. Appetite **2**, 137-143. (10.1016/S0195-6663(81)80007-4) 7039505

[14] Flatt J. 1987 The difference in the storage capacities for carbohydrate and for fat, and its implications in the regulation of body weight. Ann. N Y Acad. Sci. **499**, 104-123. (10.1111/j.1749-6632.1987.tb36202.x) 3300476

[15] Mellinkoff SM, Frankland M, Boyle D, Greipel M. 1956 Relationship between serum amino acid concentration and fluctuations in appetite. J. Appl. Physiol. **8**, 535-538. (10.1152/jappl.1956.8.5.535) 13295170

[16] Millward DJ. 1995 A protein-stat mechanism for regulation of growth and maintenance of the lean body mass. Nutr. Res. Rev. **8**, 93-120. (10.1079/NRR19950008) 19094281

[17] Raubenheimer D, Simpson SJ. 2019 Protein leverage: theoretical foundations and ten points of clarification. Obesity **27**, 1225-1238. (10.1002/oby.22531) 31339001

[18] Ohlsson C, Jansson J-O. 2020 The gravitostat theory: more data needed. EClinicalMedicine **27**, 100530. (10.1016/j.eclinm.2020.100530) 33033795 PMC7533352

[19] Cabanac M, Richard D. 1996 The nature of the ponderostat: Hervey's hypothesis revived. Appetite **26**, 45-54. (10.1006/appe.1996.0004) 8660032

[20] Hervey G. 1969 Regulation of energy balance. Nature **222**, 629-631. (10.1038/222629a0) 5768271

[21] Wirtshafter D, Davis JD. 1977 Set points, settling points, and the control of body weight. Physiol. Behav. **19**, 75-78. (10.1016/0031-9384(77)90162-7) 11803695

[22] Ludwig DS, Ebbeling CB. 2018 The carbohydrate-insulin model of obesity: beyond ‘calories in, calories out’. J. Am. Med. Assoc. Internal Med. **178**, 1098-1103. (10.1001/jamainternmed.2018.2933) PMC6082688 29971406

[23] Geiselman PJ. 1988 Sugar-induced hyperphagia: is hyperinsulinemia, hypoglycemia, or any other factor a ‘necessary’ condition? Appetite **11**, 26-34. (10.1016/S0195-6663(88)80043-6) 3056264

[24] Sclafani A. 1987 Carbohydrate-induced hyperphagia and obesity in the rat: effects of saccharide type, form, and taste. Neurosci. Biobehav. Rev. **11**, 155-162. (10.1016/S0149-7634(87)80020-9) 3302791

[25] Sclafani A. 1987 Carbohydrate taste, appetite, and obesity: an overview. Neurosci. Biobehav. Rev. **11**, 131-153. (10.1016/S0149-7634(87)80019-2) 3302790

[26] de Araujo IE, Schatzker M, Small DM. 2020 Rethinking food reward. Annu. Rev. Psychol. **71**, 139-164. (10.1146/annurev-psych-122216-011643) 31561741

[27] Li M, Tan H-E, Lu Z, Tsang KS, Chung AJ, Zuker CS. 2022 Gut–brain circuits for fat preference. Nature **610**, 722-730. (10.1038/s41586-022-05266-z) 36070796 PMC9605869

[28] Tan H-E, Sisti AC, Jin H, Vignovich M, Villavicencio M, Tsang KS, Goffer Y, Zuker CS. 2020 The gut–brain axis mediates sugar preference. Nature **580**, 511-516. (10.1038/s41586-020-2199-7) 32322067 PMC7185044

[29] Forbes JM. 2007 Voluntary food intake and diet selection in farm animals. Oxford, UK: Cabi.

[30] Stubbs R, Elia M. 2001 Macronutrients and appetite control with implications for the nutritional management of the malnourished. Clin. Nutr. **20**, 129-139. (10.1054/clnu.2001.0418)

[31] Berridge KC, Kringelbach ML. 2008 Affective neuroscience of pleasure: reward in humans and animals. Psychopharmacology (Berl.) **199**, 457-480. (10.1007/s00213-008-1099-6) 18311558 PMC3004012

[32] Hopkins M, Beaulieu K, Myers A, Gibbons C, Blundell JE. 2017 Mechanisms responsible for homeostatic appetite control: theoretical advances and practical implications. Expert Rev. Endocrinol. Metab. **12**, 401-415. (10.1080/17446651.2017.1395693) 30063436

[33] Zheng H, Berthoud H-R. 2008 Neural systems controlling the drive to eat: mind versus metabolism. Physiology **23**, 75-83. (10.1152/physiol.00047.2007) 18400690

[34] Kringelbach ML, Stein A, van Hartevelt TJ. 2012 The functional human neuroanatomy of food pleasure cycles. Physiol. Behav. **106**, 307-316. (10.1016/j.physbeh.2012.03.023) 22487544

[35] Shin AC, Zheng H, Berthoud H-R. 2009 An expanded view of energy homeostasis: neural integration of metabolic, cognitive, and emotional drives to eat. Physiol. Behav. **97**, 572-580. (10.1016/j.physbeh.2009.02.010) 19419661 PMC2765252

[36] Stussi Y, Pool ER. 2022 Multicomponential affective processes modulating food-seeking behaviors. Curr. Opin. Behav. Sci. **48**, 101226. (10.1016/j.cobeha.2022.101226)

[37] Watts AG, Kanoski SE, Sanchez-Watts G, Langhans W. 2022 The physiological control of eating: signals, neurons, and networks. Physiol. Rev. **102**, 689-813. (10.1152/physrev.00028.2020) 34486393 PMC8759974

[38] Stubbs RJ, Turicchi J. 2021 From famine to therapeutic weight loss: hunger, psychological responses, and energy balance-related behaviors. Obes. Rev. **22**, e13191. (10.1111/obr.13191) 33527688

[39] Horgan GW. 2011 The behaviour of a neutral model of weight regulated only by body mass. J. Theor. Biol. **270**, 1-6. (10.1016/j.jtbi.2010.11.001) 21078329

[40] Speakman J, Stubbs R, Mercer J. 2002 Does body mass play a role in the regulation of food intake? Proc. Nutr. Soc. **61**, 473-487. (10.1079/PNS2002194) 12691177

[41] Levitsky DA, Sewall A, Zhong Y, Barre L, Shoen S, Agaronnik N, LeClair J-L, Zhuo W, Pacanowski C. 2019 Quantifying the imprecision of energy intake of humans to compensate for imposed energetic errors: a challenge to the physiological control of human food intake. Appetite **133**, 337-343. (10.1016/j.appet.2018.11.017) 30476522

[42] Speakman JR et al. 2011 Set points, settling points and some alternative models: theoretical options to understand how genes and environments combine to regulate body adiposity. Dis. Models Mech. **4**, 733-745. (10.1242/dmm.008698) PMC3209643 22065844

[43] Polidori D, Sanghvi A, Seeley RJ, Hall KD. 2016 How strongly does appetite counter weight loss? Quantification of the feedback control of human energy intake. Obesity **24**, 2289-2295. (10.1002/oby.21653) 27804272 PMC5108589

[44] Stubbs RJ, Hopkins M, Finlayson G, Duarte C, Gibbons C, Blundell J. 2018 Potential effects of fat mass and fat-free mass on energy intake in different states of energy balance. Eur. J. Clin. Nutr. **72**, 698-709. (10.1038/s41430-018-0146-6) 29748653

[45] Kringelbach ML. 2004 Food for thought: hedonic experience beyond homeostasis in the human brain. Neuroscience **126**, 807-819. (10.1016/j.neuroscience.2004.04.035) 15207316

[46] Dhurandhar NV, Schoeller D, Brown AW, Heymsfield SB, Thomas D, Sørensen TI, Speakman JR, Jeansonne M, Allison DB. 2015 Energy balance measurement: when something is not better than nothing. Int. J. Obesity **39**, 1109-1113. (10.1038/ijo.2014.199) PMC4430460 25394308

[47] Stubbs R, Johnstone A, O'Reilly L, Poppitt S. 1998 Methodological issues relating to the measurement of food, energy and nutrient intake in human laboratory-based studies. Proc. Nutr. Soc. **57**, 357-372. (10.1079/PNS19980053) 9793992

[48] Arnold W. 2020 Seasonal differences in the physiology of wild northern ruminants. Animal **14**(S1), s124-s132. (10.1017/S1751731119003240) 32024577

[49] Young RA. 1976 Fat, energy and mammalian survival. Am. Zool. **16**, 699-710. (10.1093/icb/16.4.699)

[50] Stubbs RJ, Murgatroyd PR, Goldberg GR, Prentice AM. 1993 Carbohydrate balance and the regulation of day-to-day food intake in humans. Am. J. Clin. Nutr. **57**, 897-903. (10.1093/ajcn/57.6.897) 8503359

[51] Shetty PS, Prentice AM, Goldberg GR, Murgatroyd PR, McKenna A, Stubbs RJ, Volschenk PA. 1994 Alterations in fuel selection and voluntary food intake in response to isoenergetic manipulation of glycogen stores in humans. Am. J. Clin. Nutr. **60**, 534-543. (10.1093/ajcn/60.4.534) 8092088

[52] Kendall A, Levitsky DA, Strupp BJ, Lissner L. 1991 Weight loss on a low-fat diet: consequence of the imprecision of the control of food intake in humans. Am. J. Clin. Nutr. **53**, 1124-1129. (10.1093/ajcn/53.5.1124) 2021123

[53] Lissner L, Levitsky DA, Strupp BJ, Kalkwarf HJ, Roe DA. 1987 Dietary fat and the regulation of energy intake in human subjects. Am. J. Clin. Nutr. **46**, 886-892. (10.1093/ajcn/46.6.886) 3687822

[54] Stubbs R, Ritz P, Coward WA, Prentice AM. 1995 Covert manipulation of the ratio of dietary fat to carbohydrate and energy density: effect on food intake and energy balance in free-living men eating ad libitum. Am. J. Clin. Nutr. **62**, 330-337. (10.1093/ajcn/62.2.330) 7625339

[55] Stubbs RJ, Harbron CG, Murgatroyd PR, Prentice AM. 1995 Covert manipulation of dietary fat and energy density: effect on substrate flux and food intake in men eating ad libitum. Am. J. Clin. Nutr. **62**, 316-329. (10.1093/ajcn/62.2.316) 7625338

[56] Astrup A, Grunwald G, Melanson E, Saris W, Hill J. 2000 The role of low-fat diets in body weight control: a meta-analysis of ad libitum dietary intervention studies. Int. J. Obesity **24**, 1545-1552. (10.1038/sj.ijo.0801453) 11126204

[57] Stubbs R, Harbron C, Prentice A. 1996 Covert manipulation of the dietary fat to carbohydrate ratio of isoenergetically dense diets: effect on food intake in feeding men ad libitum. Int. J. Obesity Related Metab. Disord. J. Int. Assoc. Study Obesity **20**, 651-660. 8817359

[58] Van Stratum P, Lussenburg R, Van Wezel L, Vergroesen A, Cremer H, Agin W, Beule B, Pötter E, Kraal J. 1978 The effect of dietary carbohydrate: fat ratio on energy intake by adult women. Am. J. Clin. Nutr. **31**, 206-212. (10.1093/ajcn/31.2.206) 623041

[59] Stubbs R, Johnstone A, Harbron C, Reid C. 1998 Covert manipulation of energy density of high carbohydrate diets in ‘pseudo free-living’ humans. Int. J. Obesity **22**, 885-892. (10.1038/sj.ijo.0800676) 9756247

[60] Stubbs J, Ferres S, Horgan G. 2000 Energy density of foods: effects on energy intake. Crit. Rev. Food Sci. Nutr. **40**, 481-515. (10.1080/10408690091189248) 11186237

[61] Hall KD et al. 2022 The energy balance model of obesity: beyond calories in, calories out. Am. J. Clin. Nutr. **115**, 1243-1254. (10.1093/ajcn/nqac031) 35134825 PMC9071483

[62] Hall KD, Guyenet SJ, Leibel RL. 2018 The carbohydrate-insulin model of obesity is difficult to reconcile with current evidence. J. Am. Med. Assoc. Internal Med. **178**, 1103-1105. (10.1001/jamainternmed.2018.2920) 29971320

[63] Ludwig DS et al. 2022 Competing paradigms of obesity pathogenesis: energy balance versus carbohydrate-insulin models. Eur. J. Clin. Nutr. **76**, 1209-1221. (10.1038/s41430-022-01179-2) 35896818 PMC9436778

[64] Mazlan N, Horgan G, Whybrow S, Stubbs J. 2006 Effects of increasing increments of fat-and sugar-rich snacks in the diet on energy and macronutrient intake in lean and overweight men. Br. J. Nutr. **96**, 596-606. 16925867

[65] Whybrow S, Mayer C, Kirk TR, Mazlan N, Stubbs RJ. 2007 Effects of two weeks' mandatory snack consumption on energy intake and energy balance. Obesity **15**, 673-685. (10.1038/oby.2007.567) 17372318

[66] Malik VS, Pan A, Willett WC, Hu FB. 2013 Sugar-sweetened beverages and weight gain in children and adults: a systematic review and meta-analysis. Am. J. Clin. Nutr. **98**, 1084-1102. (10.3945/ajcn.113.058362) 23966427 PMC3778861

[67] Malik VS, Schulze MB, Hu FB. 2006 Intake of sugar-sweetened beverages and weight gain: a systematic review. Am. J. Clin. Nutr. **84**, 274-288. (10.1093/ajcn/84.1.274) 16895873 PMC3210834

[68] Goldstone AP et al. 2009 Fasting biases brain reward systems towards high-calorie foods. Eur. J. Neurosci. **30**, 1625-1635. (10.1111/j.1460-9568.2009.06949.x) 19811532

[69] Griffioen-Roose S, Mars M, Siebelink E, Finlayson G, Tome D, de Graaf C. 2012 Protein status elicits compensatory changes in food intake and food preferences. Am. J. Clin. Nutr. **95**, 32-38. (10.3945/ajcn.111.020503) 22158729 PMC3238463

[70] Bray GA, Popkin BM. 1998 Dietary fat intake does affect obesity! Am. J. Clin. Nutr. **68**, 1157-1173. (10.1093/ajcn/68.6.1157) 9846842

[71] Hooper L, Abdelhamid A, Moore HJ, Douthwaite W, Skeaff CM, Summerbell CD. 2012 Effect of reducing total fat intake on body weight: systematic review and meta-analysis of randomised controlled trials and cohort studies. Br. Med. J. **345**, e7666. (10.1136/bmj.e7666) 23220130 PMC3516671

[72] Lissner L, Heitmann BL. 1995 Dietary fat and obesity: evidence from epidemiology. Eur. J. Clin. Nutr. **49**, 79-90. 7743988

[73] Seidell JC. 1998 Dietary fat and obesity: an epidemiologic perspective. Am. J. Clin. Nutr. **67**, 546S-550S. (10.1093/ajcn/67.3.546S) 9497168

[74] Yu-Poth S, Zhao G, Etherton T, Naglak M, Jonnalagadda S, Kris-Etherton PM. 1999 Effects of the national cholesterol education program's step I and step II dietary intervention programs on cardiovascular disease risk factors: a meta-analysis. Am. J. Clin. Nutr. **69**, 632-646. (10.1093/ajcn/69.4.632) 10197564

[75] McCance RA, Widdowson EM. 2014 Mccance and Widdowson's the composition of foods. London, UK: Royal Society of Chemistry.

[76] Holland B, Welch A, Unwin I, Buss D, Paul A, Southgate D. 1991 Mccance and Widdowson's the composition of foods. London, UK: Royal Society of Chemistry.

[77] Bates B, Lennox A, Prentice A, Bates CPP, a N.S.e. 2014 Nutrition Survey results from years 1, 2, 3 and 4 (combined) of the Rolling Programme (2008/2009–2011/2012). London, UK: Public Health England.

[78] Page P, Steer T, Bates B, Cox LJ, Nicolson S, Prentice A, Swan G. 2016 National Diet and Nutrition Survey results from years 5 and 6 (combined) of the Rolling Programme (2012/2013–2013/2014). London, UK: Public Health England.

[79] Ramirez I, Tordoff MG, Friedman MI. 1989 Dietary hyperphagia and obesity: what causes them? Physiol. Behav. **45**, 163-168. (10.1016/0031-9384(89)90180-7) 2657817

[80] Stubbs R, Johnstone A, O'Reilly L, Barton K, Reid C. 1998 The effect of covertly manipulating the energy density of mixed diets on ad libitum food intake in ‘pseudo free-living’ humans. Int. J. Obesity **22**, 980-987. (10.1038/sj.ijo.0800715) 9806313

[81] Walls EK, Koopmans HS. 1992 Differential effects of intravenous glucose, amino acids, and lipid on daily food intake in rats. Amer. J. Physiol. Regul. Integr. Comp. Physiol. **262**, R225-R234. (10.1152/ajpregu.1992.262.2.R225) 1539730

[82] Gil KM, Skeie B, Kvetan V, Askanazi J, Friedman MI. 1991 Parenteral nutrition and oral intake: effect of glucose and fat infusions. J. Parent. Enteral Nutr. **15**, 426-432. (10.1177/0148607191015004426) 1910106

[83] de Castro JM. 1998 Prior day's intake has macronutrient-specific delayed negative feedback effects on the spontaneous food intake of free-living humans. J. Nutr. **128**, 61-67. (10.1093/jn/128.1.61) 9430603

[84] Stubbs R. 1995 Macronutrient effects on appetite. Int. J. Obesity **19**, S11-S19. 8581107

[85] Weststrate J. 1992 Effect of nutrients on the regulation of food intake. Vlaardingen, The Netherlands: Unilever Research.

[86] Barkeling B, Rössner S, Björvell H. 1990 Effects of a high-protein meal (meat) and a high-carbohydrate meal (vegetarian) on satiety measured by automated computerized monitoring of subsequent food intake, motivation to eat and food preferences. Int. J. Obesity **14**, 743-751. 2228407

[87] Hill AJ, Blundell JE. 1986 Macronutrients and satiety: the effects of a high-protein or high-carbohydrate meal on subjective motivation to eat and food preferences. Nutr. Behav. (USA) **3**, 133-144.

[88] Hill AJ, Blundell JE. 1989 Comparison of the action of macronutrients on the expression of appetite in lean and obese human subjects. Ann. N Y Acad. Sci. (USA) **575**, 529-531. (10.1111/j.1749-6632.1989.tb53283.x)

[89] Latner J, Schwartz M. 1999 The effects of a high-carbohydrate, high-protein or balanced lunch upon later food intake and hunger ratings. Appetite **33**, 119-128. (10.1006/appe.1999.0237) 10447984

[90] Poppitt SD, McCormack D, Buffenstein R. 1998 Short-term effects of macronutrient preloads on appetite and energy intake in lean women. Physiol. Behav. **64**, 279-285. (10.1016/S0031-9384(98)00061-4) 9748094

[91] Johnstone A, Stubbs R, Harbron C. 1996 Effect of overfeeding macronutrients on day-to-day food intake in man. Eur. J. Clin. Nutr. **50**, 418-430. 8862477

[92] Cotton JR, Burley VJ, Weststrate JA, Blundell JE. 2007 Dietary fat and appetite: similarities and differences in the satiating effect of meals supplemented with either fat or carbohydrate. J. Hum. Nutr. Diet. **20**, 186-199. (10.1111/j.1365-277X.2007.00769.x) 17539869

[93] Rolls BJ, Kim-Harris S, Fischman MW, Foltin RW, Moran TH, Stoner SA. 1994 Satiety after preloads with different amounts of fat and carbohydrate: implications for obesity. Am. J. Clin. Nutr. **60**, 476-487. (10.1093/ajcn/60.4.476) 7661908

[94] Robinson E, Khuttan M, Patel Z, Jones A. 2022 Calorie reformulation: a systematic review and meta-analysis examining the effect of manipulating food energy density on daily energy intake. Int. J. Behav. Nutr. Phys. Activity **19**, 1-19. (10.1186/s12966-022-01287-z) PMC9026919 35459185

[95] Drewnowski A. 1989 Sensory preferences for fat and sugar in adolescence and adult life. *Ann. N Y Acad. Sci*. **561**, 243-250. (10.1111/j.1749-6632.1989.tb20986.x) 2735681

[96] Gerstein DE, Woodward-Lopez G, Evans AE, Kelsey K, Drewnowski A. 2004 Clarifying concepts about macronutrients' effects on satiation and satiety. J. Am. Diet Assoc. **104**, 1151-1153. (10.1016/j.jada.2004.04.027) 15215775

[97] Drewnowski A, Krahn DD, Demitrack MA, Nairn K, Gosnell BA. 1992 Taste responses and preferences for sweet high-fat foods: evidence for opioid involvement. Physiol. Behav. **51**, 371-379. (10.1016/0031-9384(92)90155-U) 1313591

[98] DiFeliceantonio AG, Coppin G, Rigoux L, Thanarajah SE, Dagher A, Tittgemeyer M, Small DM. 2018 Supra-additive effects of combining fat and carbohydrate on food reward. Cell Metab. **28**, 33-44. e33. (10.1016/j.cmet.2018.05.018) 29909968

[99] Benelam B. 2009 Satiation, satiety and their effects on eating behaviour. Nutr. Bull. **34**, 126-173. (10.1111/j.1467-3010.2009.01753.x)

[100] Robinson E, Horgan G, Stubbs J. 2023 Convincing experimental data is required to revisit the passive overconsumption hypothesis. Amer. J. Clin. Nutr. **117**, 635-636. (10.1016/j.ajcnut.2022.09.003) 36872022

[101] Contreras-Rodriguez O, Solanas M, Escorihuela RM. 2022 Dissecting ultra-processed foods and drinks: do they have a potential to impact the brain? Rev. Endocr. Metab. Disorders **23**, 697-717. (10.1007/s11154-022-09711-2) 35107734

[102] Hall KD et al. 2019 Ultra-processed diets cause excess calorie intake and weight gain: an inpatient randomized controlled trial of ad libitum food intake. Cell Metab. **30**, 67-77. e63. (10.1016/j.cmet.2019.05.008) 31105044 PMC7946062

[103] LaFata EM, Gearhardt AN. 2022 Ultra-processed food addiction: an epidemic? Psychother. Psychosom. **91**, 363-372. (10.1159/000527322) 36349805

[104] Ludwig DS, Astrup A, Bazzano LA, Ebbeling CB, Heymsfield SB, King JC, Willett WC. 2019 Ultra-processed food and obesity: the pitfalls of extrapolation from short studies. Cell Metab. **30**, 3-4. (10.1016/j.cmet.2019.06.004) 31230987

[105] Pagliai G, Dinu M, Madarena M, Bonaccio M, Iacoviello L, Sofi F. 2021 Consumption of ultra-processed foods and health status: a systematic review and meta-analysis. Br. J. Nutr. **125**, 308-318. (10.1017/S0007114520002688) 32792031 PMC7844609

[106] Rolls BJ, Cunningham PM, Diktas HE. 2020 Properties of ultraprocessed foods that can drive excess intake. Nutr. Today **55**, 109-115. (10.1097/NT.0000000000000410)

[107] Steele EM, Raubenheimer D, Simpson SJ, Baraldi LG, Monteiro CA. 2018 Ultra-processed foods, protein leverage and energy intake in the USA. Public Health Nutr. **21**, 114-124. (10.1017/S1368980017001574) 29032787 PMC10260799

[108] Tobias DK, Hall KD. 2021 Eliminate or reformulate ultra-processed foods? Biological mechanisms matter. Cell Metab. **33**, 2314-2315. (10.1016/j.cmet.2021.10.005) 34699743

[109] Visioli F et al. In press. The ultra-processed foods hypothesis: a product processed well beyond the basic ingredients in the package. Nutr. Res. Rev. 1-31. (10.1017/S0954422422000117) 35730561

[110] Gibney MJ, Forde CG. 2022 Nutrition research challenges for processed food and health. Nat. Food **3**, 104-109. (10.1038/s43016-021-00457-9) 37117956

[111] Moubarac J-C, Parra DC, Cannon G, Monteiro CA. 2014 Food classification systems based on food processing: significance and implications for policies and actions: a systematic literature review and assessment. Curr. Obesity Rep. **3**, 256-272. (10.1007/s13679-014-0092-0) 26626606

[112] Cohen DA, Babey SH. 2012 Contextual influences on eating behaviours: heuristic processing and dietary choices. Obes. Rev. **13**, 766-779. (10.1111/j.1467-789X.2012.01001.x) 22551473 PMC3667220

[113] Van't Riet J, Sijtsema SJ, Dagevos H, De Bruijn G-J. 2011 The importance of habits in eating behaviour. An overview and recommendations for future research. Appetite **57**, 585-596. (10.1016/j.appet.2011.07.010) 21816186

[114] Caballero B. 2005 Encyclopedia of human nutrition. Amsterdam, The Netherlands: Elsevier.

[115] Stubbs R, Whybrow S. 2004 Energy density, diet composition and palatability: influences on overall food energy intake in humans. Physiol. Behav. **81**, 755-764. (10.1016/j.physbeh.2004.04.027) 15234181

[116] Stubbs J, Blundell JE. 2003 Diet composition and the control of food intake in humans. In Handbook of obesity: etiology and pathophysiology, vol. 3 (eds GA Bray, C Bouchard), pp. 443-476. New York, NY: CRC Press.

[117] Livingstone MBE, Black AE. 2003 Markers of the validity of reported energy intake. J. Nutr. **133**, 895S-920S. (10.1093/jn/133.3.895S) 12612176

[118] Jobarteh ML et al. 2020 Development and validation of an objective, passive dietary assessment method for estimating food and nutrient intake in households in low-and middle-income countries: a study protocol. Curr. Dev. Nutr. **4**, nzaa020. (10.1093/cdn/nzaa020) 32099953 PMC7031207

[119] Lei J, Qiu J, Lo FP-W, Lo B. 2021 Assessing individual dietary intake in food sharing scenarios with food and human pose detection. In Pattern Recognition ICPR Int. Workshops and Challenges: virtual event, 10–15 January 2021, Proc., Part V, pp. 549-557. Berlin, Germany: Springer.

[120] Lo FPW, Sun Y, Qiu J, Lo B. 2020 Image-based food classification and volume estimation for dietary assessment: a review. IEEE J. Biomed. Health Inform. **24**, 1926-1939. (10.1109/JBHI.2020.2987943) 32365038

[121] Qiu J, Lo FP-W, Jiang S, Tsai Y-Y, Sun Y, Lo B. 2020 Counting bites and recognizing consumed food from videos for passive dietary monitoring. IEEE J. Biomed. Health Inform. **25**, 1471-1482. (10.1109/JBHI.2020.3022815) 32897866

[122] O'Driscoll R, Turicchi J, Hopkins M, Duarte C, Horgan GW, Finlayson G, Stubbs RJ. 2021 Comparison of the validity and generalizability of machine learning algorithms for the prediction of energy expenditure: validation study. JMIR mHealth and uHealth **9**, e23938. (10.2196/23938) 34346890 PMC8374660

[123] O'Driscoll R, Turicchi J, Hopkins M, Horgan GW, Finlayson G, Stubbs JR. 2020 Improving energy expenditure estimates from wearable devices: a machine learning approach. J. Sports Sci. **38**, 1496-1505. (10.1080/02640414.2020.1746088) 32252598

[124] White T, Westgate K, Hollidge S, Venables M, Olivier P, Wareham N, Brage S. 2019 Estimating energy expenditure from wrist and thigh accelerometry in free-living adults: a doubly labelled water study. Int. J. Obesity **43**, 2333-2342. (10.1038/s41366-019-0352-x) PMC7358076 30940917

[125] White T, Westgate K, Wareham NJ, Brage S. 2016 Estimation of physical activity energy expenditure during free-living from wrist accelerometry in UK adults. PLoS ONE **11**, e0167472. 27936024 10.1371/journal.pone.0167472 PMC5147924

[126] Zhu J, Pande A, Mohapatra P, Han JJ. 2015 Using deep learning for energy expenditure estimation with wearable sensors. In 17th Int. Conf. on E-health Networking, Application & Services (HealthCom), pp. 501-506. Piscataway, NJ: IEEE.

[127] Drewnowski A, Buszkiewicz J, Aggarwal A, Rose C, Gupta S, Bradshaw A. 2020 Obesity and the built environment: a reappraisal. Obesity **28**, 22-30. (10.1002/oby.22672) 31782242 PMC6986313

[128] Fillekes MP, Giannouli E, Kim E-K, Zijlstra W, Weibel R. 2019 Towards a comprehensive set of GPS-based indicators reflecting the multidimensional nature of daily mobility for applications in health and aging research. Int. J. Health Geogr. **18**, 1-20. (10.1186/s12942-019-0181-0) 31340812 PMC6657041

[129] Jerrett M, Gale S, Kontgis C. 2010 Spatial modeling in environmental and public health research. Int. J. Environ. Res. Public Health **7**, 1302-1329. (10.3390/ijerph7041302) 20617032 PMC2872363

[130] Dakin C, Beaulieu K, Hopkins M, Gibbons C, Finlayson G, Stubbs RJ. 2023 Do eating behavior traits predict energy intake and body mass index? A systematic review and meta-analysis. Obes. Rev. **24**, e13515. (10.1111/obr.13515) 36305739 PMC10078190

[131] Vainik U, García-García I, Dagher A. 2019 Uncontrolled eating: a unifying heritable trait linked with obesity, overeating, personality and the brain. Eur. J. Neurosci. **50**, 2430-2445. (10.1111/ejn.14352) 30667547

[132] Berthoud H-R, Morrison CD, Ackroff K, Sclafani A. 2021 Learning of food preferences: mechanisms and implications for obesity & metabolic diseases. Int. J. Obesity **45**, 2156-2168. (10.1038/s41366-021-00894-3) PMC8455326 34230576

